# Purpureocillium lilacinum Pneumonia in Myositis-Associated Interstitial Lung Disease: Successful Treatment With Oral Itraconazole

**DOI:** 10.7759/cureus.112954

**Published:** 2026-07-19

**Authors:** Muhammad Naimmuddin Abdul Azih, Wan Nurliyana Wan Ramli, Nurul Aulia Zakaria

**Affiliations:** 1 Internal Medicine/Pulmonology, International Islamic University Malaysia, Kuantan, MYS; 2 Internal Medicine/Infectious Diseases, International Islamic University Malaysia, Kuantan, MYS; 3 Rheumatology, International Islamic University Malaysia, Kuantan, MYS

**Keywords:** anti-jo1 antibody, ground-glass opacities, hyalohyphomycosis, itraconazole, mofetil mycophenolate, myositis-related interstitial lung disease, purpureocillium lilacinum pneumonia

## Abstract

*Purpureocillium lilacinum* (*P. lilacinum*) is a rare opportunistic mold that predominantly affects immunocompromised hosts and exhibits intrinsic resistance to several conventional antifungal agents. Pulmonary infection is uncommon, and only a limited number of cases have been reported in patients with autoimmune connective tissue diseases receiving immunosuppressive therapy. We report a 37-year-old male hospital social worker with anti-Jo-1-positive myositis-associated interstitial lung disease receiving corticosteroids and mycophenolate mofetil who presented with hemoptysis and dyspnea. Computed tomography of the thorax demonstrated bilateral ground-glass opacities, multilobar consolidation, tree-in-bud nodularity, and mediastinal lymphadenopathy. Bronchoalveolar lavage fungal culture yielded *P. lilacinum*, with species identification confirmed by matrix-assisted laser desorption/ionization time-of-flight mass spectrometry (MALDI-TOF MS). Because second-generation triazoles were unavailable at the time of diagnosis, treatment was initiated with oral itraconazole (200 mg twice daily) together with temporary cessation of immunosuppressive therapy. The patient experienced rapid clinical improvement, with resolution of the acute pulmonary infiltrates on follow-up computed tomography after three months and a negative follow-up sputum fungal culture after six months of therapy. Immunosuppressive treatment was subsequently reintroduced without recurrence of infection or progression of interstitial lung disease during follow-up. This case highlights the diagnostic challenge of probable pulmonary *P. lilacinum* infection in immunosuppressed patients with myositis-associated interstitial lung disease. It demonstrates a favorable clinical course during prolonged oral itraconazole therapy when preferred antifungal agents were unavailable. However, treatment outcomes should be interpreted with caution, as this is a single case, and concomitant antibacterial therapy, along with temporary modification of immunosuppressive therapy, may also have contributed to recovery.

## Introduction

*Purpureocillium lilacinum* (*P. lilacinum*) is an emerging opportunistic filamentous fungus that has increasingly been recognized as a cause of invasive infection in immunocompromised hosts. Although commonly found in soil and decaying vegetation, it rarely causes disease in humans. Reported manifestations include cutaneous, ocular, sinus, musculoskeletal, and disseminated infections, with pulmonary involvement being particularly uncommon [1-8].

The diagnosis of pulmonary *P. lilacinum* infection remains challenging because its clinical presentation and radiological features are non-specific and may mimic bacterial pneumonia, invasive aspergillosis, mycobacterial infection, or progression of the underlying interstitial lung disease (ILD) [3,5,7]. Accurate species identification is particularly important because *P. lilacinum* exhibits intrinsic resistance to several conventional antifungal agents, including amphotericin B and fluconazole, while demonstrating variable susceptibility among triazole antifungal agents [1,3]. Laboratory identification using validated methods, such as matrix-assisted laser desorption/ionization time-of-flight mass spectrometry (MALDI-TOF MS) or molecular techniques where available, facilitates timely diagnosis and appropriate selection of antifungal therapy.

Patients with idiopathic inflammatory myopathies and myositis-associated ILD frequently require prolonged immunosuppressive therapy, predisposing them to opportunistic infections [3]. In this population, distinguishing pulmonary infection from worsening ILD may be difficult because both conditions can present with respiratory symptoms and new pulmonary infiltrates. Consequently, delayed recognition may result in inappropriate treatment and adverse outcomes.

Pulmonary *P. lilacinum* infection has rarely been reported in patients with myositis-associated ILD. We report a case of probable *P. lilacinum* pneumonia in a patient with anti-Jo-1-positive myositis-associated ILD who was receiving corticosteroids and mycophenolate mofetil (MMF), highlighting the diagnostic challenges and the favorable clinical and radiological outcomes achieved with prolonged oral itraconazole therapy.

This article was previously presented as a meeting abstract at the 20th Asia Pacific Congress of Clinical Microbiology and Infection (APCCMI 2025) in Bangkok, Thailand, in December 2025.

## Case presentation

A 37-year-old male hospital social worker with a prior diagnosis of anti-Jo-1-positive myositis-associated ILD, treated initially with tapering corticosteroids, followed by MMF as a steroid-sparing agent, presented with a one-week history of hemoptysis and exertional dyspnea. His medical history included obesity, obstructive sleep apnea, hypertension, type 2 diabetes mellitus, dyslipidemia, heart failure, and pulmonary hypertension. Despite these comorbidities, he had remained clinically stable with only mild exertional dyspnea and continued working without functional limitation.

**Figure 1:**
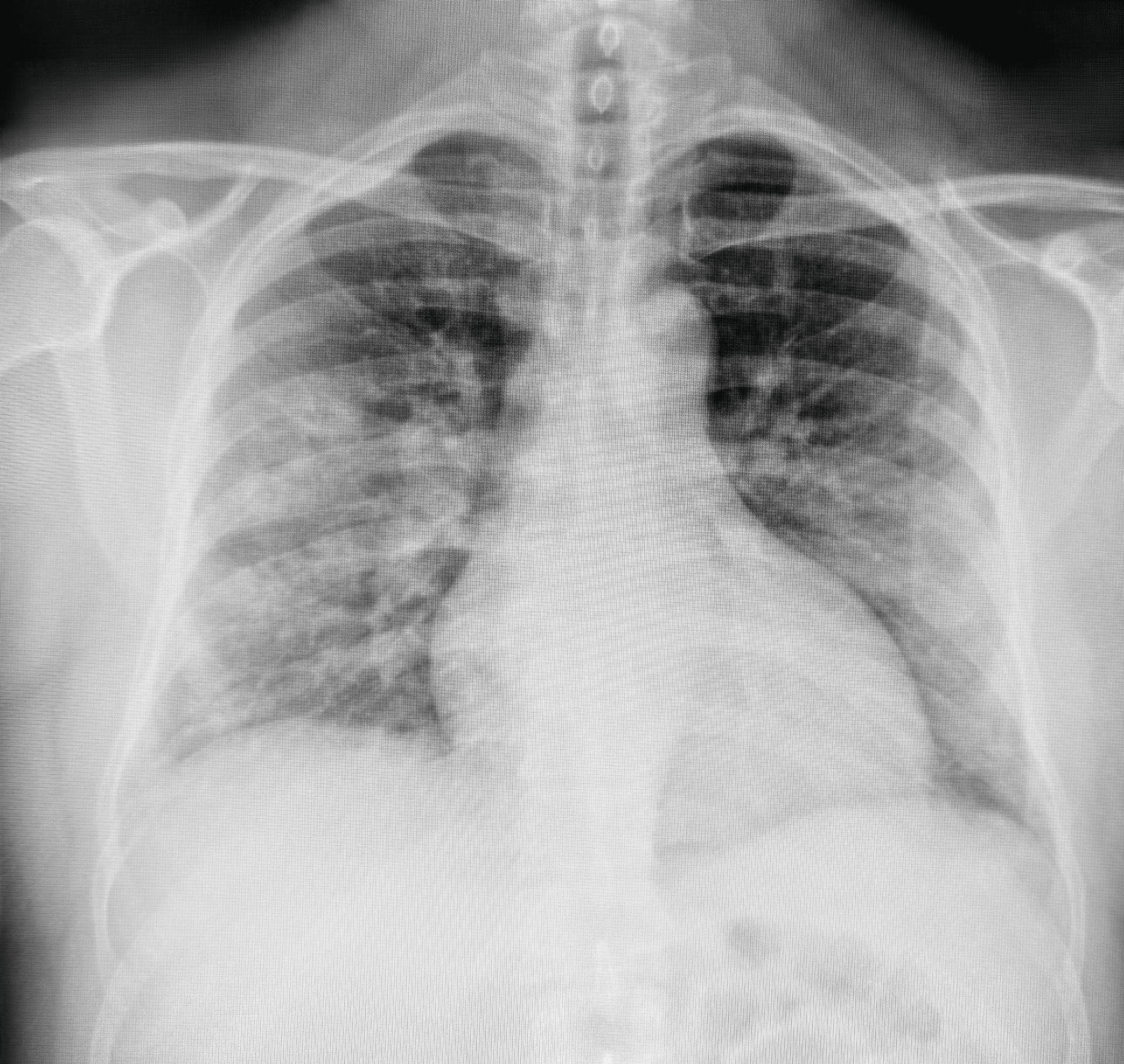
Chest radiograph Bilateral non-specific lung opacities with cardiomegaly.

**Figure 2:**
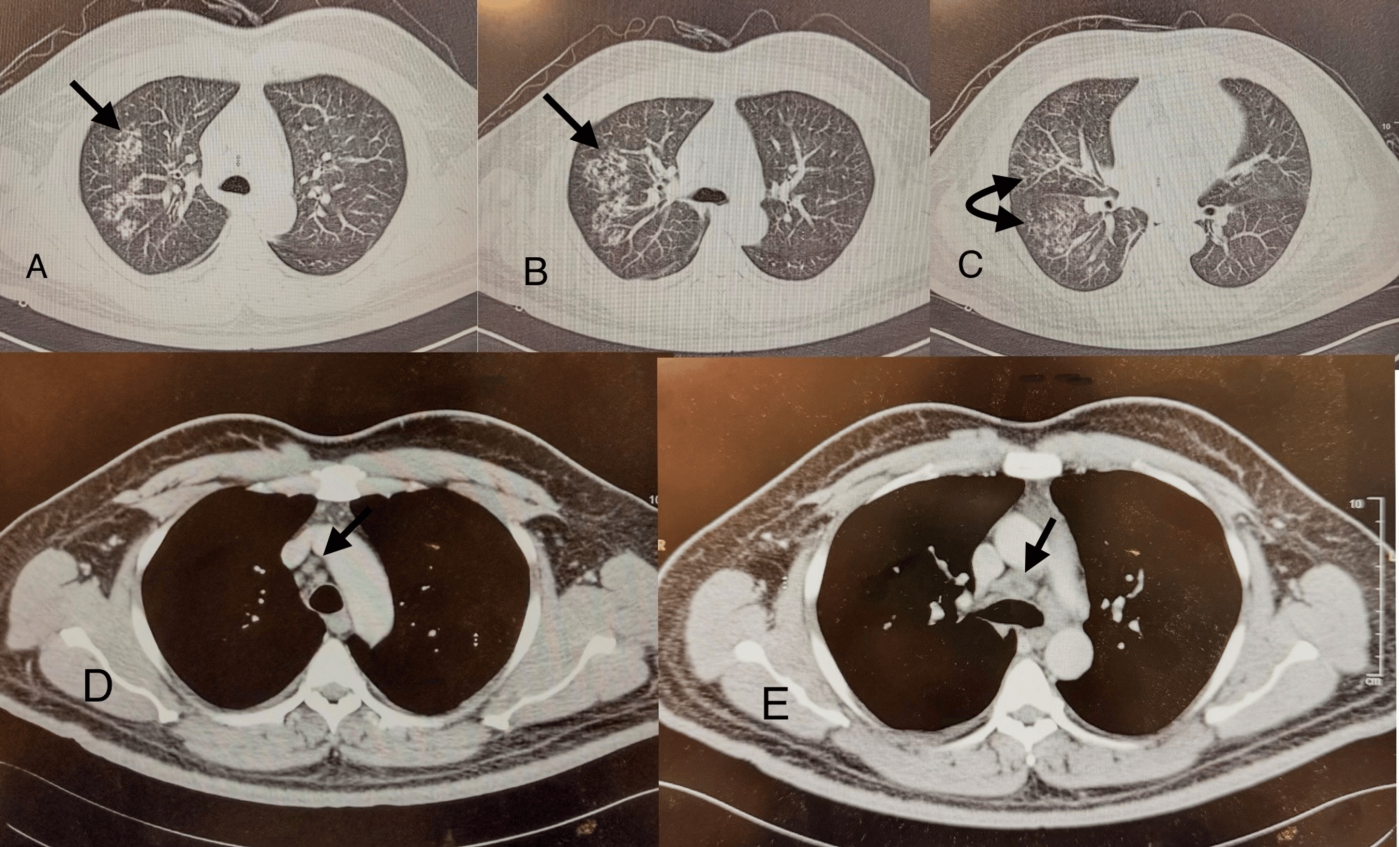
Contrast-enhanced CT thorax before treatment (A, B, and C) Contrast-enhanced CT thorax before treatment. Axial CT images in the lung window demonstrate patchy consolidation seen in the right upper, middle, and lower lobes (straight black arrows in A and B), with a tree-in-bud lesion and ground-glass opacities highlighted by curved black arrows in (C). (D and E) Axial CT images in mediastinal window reveal enlarged mediastinal lymph nodes. The straight black arrow in (D) indicates enlarged nodes seen in the pre-vascular and paratracheal regions, and the straight black arrow in (E) highlights an enlarged node in the pre-carinal region. CT: computed tomography

During optimization of MMF therapy, he developed these new respiratory symptoms. Chest radiography demonstrated bilateral non-specific pulmonary opacities with cardiomegaly (Figure 1). He remained hemodynamically stable and required supplemental oxygen via nasal cannula at 3 L/min. Empirical treatment for community-acquired pneumonia was initiated with intravenous ceftriaxone and oral azithromycin. On the same day, contrast-enhanced computed tomography of the thorax demonstrated extensive bilateral ground-glass opacities, multilobar consolidation, tree-in-bud nodularity, and mediastinal lymphadenopathy (Figure 2), findings considered compatible with pulmonary infection in the appropriate clinical context.

Given the differential diagnoses of diffuse alveolar hemorrhage, pulmonary tuberculosis, and opportunistic pulmonary infection, urgent flexible bronchoscopy with bronchoalveolar lavage (BAL) was performed. BAL returned hemorrhagic fluid; however, the sequential lavage appearance did not become progressively bloodier to suggest diffuse alveolar hemorrhage, and cytological evidence of hemosiderin-laden macrophages was negative. No endobronchial abnormalities were identified. Investigations for pulmonary tuberculosis and a respiratory pathogen panel (36 PCR targets, including *Pneumocystis jirovecii* PCR) were negative. BAL fungal culture subsequently yielded *P. lilacinum*, with species identification confirmed by MALDI-TOF MS. In view of the patient’s immunosuppressed status, compatible clinical presentation, radiological findings, and subsequent therapeutic response, this microbiological finding was considered most consistent with probable pulmonary *P. lilacinum* infection rather than airway colonization or laboratory contamination.

Because second-generation triazoles were unavailable at the time of diagnosis, oral itraconazole 200 mg twice daily (total daily dose of 400 mg) was initiated following multidisciplinary discussion with the infectious diseases team and the patient. The selected dose was based on published reports that demonstrate variable susceptibility and relatively high minimum inhibitory concentrations (MICs) of itraconazole against *P. lilacinum*. Antifungal susceptibility testing was not performed because a standardized method for this organism was unavailable at the laboratory performing the testing. Immunosuppressive therapy was temporarily withheld. Although the patient had also received ceftriaxone and azithromycin for presumed community-acquired pneumonia, hemoptysis persisted despite five days of empirical antibacterial therapy. It only began to resolve several days after oral itraconazole was initiated. His oxygen requirements progressively improved, and he tolerated treatment well without clinically significant adverse effects. Serial liver function tests remained within normal limits throughout therapy, and his cardiac status remained clinically stable.

**Figure 3:**
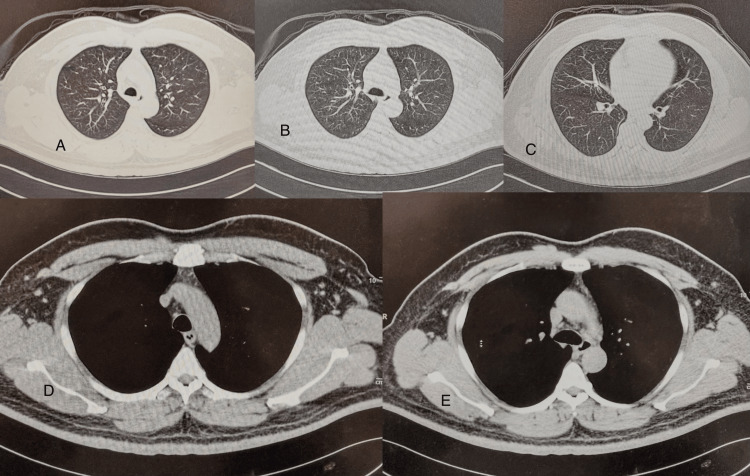
Contrast-enhanced CT thorax after treatment (A, B, and C) Follow-up CT images in the lung window demonstrate complete resolution of the previous lesions. (D and E) Follow-up axial CT images in the mediastinal window show complete resolution or significant regression of previously noted mediastinal lymphadenopathy, with the paratracheal (D) and precarinal lymph nodes (E) returning to normal size. CT: computed tomography

The patient was discharged after two weeks with continued oral itraconazole therapy. Owing to the marked clinical response, the decision was made to continue itraconazole rather than switch to voriconazole when it became available later. Follow-up contrast-enhanced CT thorax performed three months after treatment initiation demonstrated complete resolution of the previously described acute pulmonary infiltrates and significant regression of mediastinal lymphadenopathy (Figure 3). After six months of oral itraconazole 200 mg twice daily (400 mg/day), follow-up sputum fungal culture was negative, and antifungal therapy was discontinued. Although a negative sputum culture alone does not establish microbiological eradication of lower respiratory tract infection, the absence of recurrent respiratory symptoms together with sustained clinical and radiological improvement supported successful treatment.

MMF was reintroduced three months later under rheumatology supervision, and the patient’s myositis-associated ILD remained clinically stable without evidence of recurrent fungal infection. Therapeutic drug monitoring for itraconazole was not performed. Nevertheless, serial liver function tests remained normal, no clinically significant drug-related adverse effects were observed, and the patient’s underlying heart failure remained clinically stable throughout treatment and follow-up. Transthoracic echocardiography performed at two, four, and six months demonstrated stable cardiac function without evidence of deterioration during itraconazole treatment. Pulmonary function testing performed at six months showed an FEV1 of 70% predicted, an FVC of 75% predicted, and an FEV1/FVC ratio of 0.85, consistent with stable underlying myositis-associated ILD. The six-minute walk test also remained within normal limits, and he continues to work without functional limitation. He remained asymptomatic for over one year during routine follow-up.

## Discussion

This appears to be a rare reported case of probable pulmonary *P. lilacinum* infection in a patient with anti-Jo-1-positive myositis-associated ILD receiving immunosuppressive therapy who experienced favorable clinical and radiological improvement during prolonged oral itraconazole therapy. The diagnosis was supported by compatible clinical and radiological findings, BAL fungal culture with species identification confirmed by MALDI-TOF MS, and subsequent sustained clinical and radiological improvement following antifungal therapy. Histopathological confirmation, antifungal susceptibility testing, and therapeutic drug monitoring were unavailable in this case. Furthermore, because immunosuppressive therapy was temporarily withheld and empirical antibacterial therapy with ceftriaxone and azithromycin was administered before itraconazole, the favorable outcome should be interpreted in the context of the overall clinical course rather than as definitive evidence of itraconazole efficacy alone.

Fungal infections are recognized as an important cause of opportunistic pulmonary infections in patients with idiopathic inflammatory myopathies, particularly those with associated ILD. In a cohort of 279 patients with inflammatory myopathy, fungi accounted for approximately 40% of opportunistic pulmonary infections, with *Pneumocystis jirovecii* and *Candida albicans* being the most frequently identified pathogens. Among patients with anti-MDA5 antibody-positive ILD, fungal infections have been associated with substantially increased mortality, with one study reporting a significantly higher three-month mortality rate than in patients without fungal infection (85.7% vs. 38.4%) [2]. Usuda et al. further highlighted the clinical significance of pulmonary *P. lilacinum* infection, reporting pulmonary involvement in approximately 26% of invasive cases, predominantly among immunocompromised patients, with an overall mortality rate of 22% and nearly half of these deaths directly attributable to fungal infection. The same review also reported poorer outcomes among patients treated with amphotericin B, emphasizing the importance of selecting an appropriate triazole antifungal when feasible [3].

The clinical manifestations of pulmonary *P. lilacinum* infection are non-specific, with fever being the most frequently reported symptom. Hemoptysis has been described in association with cavitary pulmonary disease and invasive fungal infection, including patients presenting with progressive hemoptysis and exertional dyspnea [4]. In immunocompromised patients with underlying ILD, hemoptysis may also create diagnostic uncertainty because pulmonary infection, diffuse alveolar hemorrhage, acute exacerbation of ILD, and pulmonary tuberculosis may present with similar clinical features [5]. Consequently, a high index of clinical suspicion is required to avoid delayed diagnosis and inappropriate treatment.

The diagnosis of pulmonary *P. lilacinum* infection requires integration of host risk factors, compatible clinical and radiological findings, and mycological evidence obtained from respiratory specimens. Although BAL fungal culture remains an important diagnostic investigation, isolation of *P. lilacinum* alone does not distinguish true pulmonary infection from airway colonization or laboratory contamination. Characteristic microbiological findings include floccose lilac-colored colonies on Sabouraud dextrose agar and microscopic demonstration of characteristic verticillate conidiophores with swollen-based phialides arranged in brush-like structures [3]. Because morphological features may overlap with those of other filamentous fungi, species-level identification using validated methods, including MALDI-TOF MS or molecular techniques where appropriate, may improve diagnostic confidence [6]. In the present case, the diagnosis of probable pulmonary infection was supported by the patient’s immunosuppressed status, compatible CT findings, MALDI-TOF-confirmed BAL isolate, and sustained clinical and radiological improvement during antifungal therapy. Histopathological confirmation was unavailable, and antifungal susceptibility testing could not be performed because a standardized method for this organism was not available in the performing laboratory.

Radiological manifestations of pulmonary *P. lilacinum* infection are non-specific and highly variable, frequently resembling bacterial pneumonia, invasive aspergillosis, mycobacterial infection, or other opportunistic pulmonary infections [3,7]. Reported CT findings include pulmonary nodules, nodular infiltrates, multilobar consolidation, the halo sign, cavitary lesions, tree-in-bud nodularity, centrilobular nodules, and pleural effusions [3-7]. These imaging findings should therefore be interpreted in conjunction with the clinical presentation and microbiological evidence rather than considered diagnostic in isolation. In our patient, extensive bilateral ground-glass opacities, multilobar consolidation, tree-in-bud nodularity, and mediastinal lymphadenopathy were considered compatible with pulmonary infection within the appropriate clinical context, and the acute radiological abnormalities subsequently resolved following treatment.

Management of pulmonary *P. lilacinum* infection is challenging because of the organism’s intrinsic resistance to several conventional antifungal agents, particularly amphotericin B and fluconazole [1,6]. Available clinical experience and susceptibility data generally favor second-generation triazoles, particularly voriconazole and posaconazole; however, treatment should be individualized based on isolate susceptibility when available, infection severity, drug availability, toxicity profile, and host-related factors, rather than relying solely on in vitro MICs [1]. More recently, isavuconazole has also demonstrated favorable outcomes in selected patients intolerant of voriconazole [6].

Although itraconazole is generally considered less active due to variable susceptibility and occasionally elevated MICs, successful outcomes have been reported in selected patients, particularly when combined with a reduction in immunosuppressive therapy and prolonged treatment durations of more than 12 weeks [3,8]. In the largest global registry of 101 reported cases, itraconazole was the second most frequently prescribed antifungal agent (28.9%), often used as oral step-down therapy or when preferred antifungal agents were unavailable [1]. In the present case, oral itraconazole was selected because second-generation triazoles were unavailable at the time of diagnosis. The patient demonstrated sustained clinical and radiological improvement without clinically significant adverse effects during treatment. Nevertheless, broader conclusions regarding itraconazole efficacy should be interpreted with caution because this is a single case, antifungal susceptibility testing and therapeutic drug monitoring were unavailable, and the temporary withdrawal of immunosuppressive therapy, together with concomitant antibacterial treatment, may also have contributed to the favorable outcome.

## Conclusions

*P. lilacinum* should be considered as a potential opportunistic pulmonary pathogen in immunosuppressed patients with myositis-associated interstitial lung disease who develop new respiratory symptoms and pulmonary infiltrates. Because the clinical and radiological manifestations are non-specific and may mimic pulmonary tuberculosis, bacterial pneumonia, diffuse alveolar hemorrhage, or deterioration of the underlying ILD, diagnosis requires integration of host risk factors, compatible clinical and radiological findings, and mycological evidence from respiratory specimens, with species-level identification using validated laboratory methods where available.

This case illustrates a favorable clinical and radiological outcome during prolonged oral itraconazole therapy in a patient with probable pulmonary *P. lilacinum* infection. Although second-generation triazoles remain the preferred treatment when available, itraconazole may represent a reasonable alternative in carefully selected patients when preferred agents are unavailable. However, the contribution of concomitant antibacterial therapy and temporary modification of immunosuppressive treatment should also be taken into consideration, and broader therapeutic conclusions cannot be drawn from a single case.
